# Conservation Efforts May Increase Malaria Burden in the Brazilian Amazon

**DOI:** 10.1371/journal.pone.0057519

**Published:** 2013-03-06

**Authors:** Denis Valle, James Clark

**Affiliations:** 1 University Program in Ecology, Duke University, Durham, North Carolina, United States of America; 2 Nicholas School of the Environment, Department of Biology, Department of Statistical Science, Duke University, Durham, North Carolina, United States of America; Tulane University School of Public Health and Tropical Medicine, United States of America

## Abstract

**Background:**

Large-scale forest conservation projects are underway in the Brazilian Amazon but little is known regarding their public health impact. Current literature emphasizes how land clearing increases malaria incidence, leading to the conclusion that forest conservation decreases malaria burden. Yet, there is also evidence that proximity to forest fringes increases malaria incidence, which implies the *opposite* relationship between forest conservation and malaria. We compare the effect of these environmental factors on malaria and explore its implications.

**Methods and Findings:**

Using a large malaria dataset (∼1,300,000 positive malaria tests collected over ∼4.5 million km^2^), satellite imagery, permutation tests, and hierarchical Bayesian regressions, we show that greater forest cover (as a proxy for proximity to forest fringes) tends to be associated with higher malaria incidence, and that forest cover effect was 25 times greater than the land clearing effect, the often cited culprit of malaria in the region. These findings have important implications for land use/land cover (LULC) policies in the region. We find that cities close to protected areas (PA’s) tend to have higher malaria incidence than cities far from PA’s. Using future LULC scenarios, we show that avoiding 10% of deforestation through better governance might result in an average 2-fold increase in malaria incidence by 2050 in urban health posts.

**Conclusions:**

Our results suggest that cost analysis of reduced carbon emissions from conservation efforts in the region should account for increased malaria morbidity, and that conservation initiatives should consider adopting malaria mitigation strategies. Coordinated actions from disparate science fields, government ministries, and global initiatives (e.g., Reduced Emissions from Deforestation and Degradation; Millenium Development Goals; Roll Back Malaria; and Global Fund to Fight AIDS, Tuberculosis and Malaria), will be required to decrease malaria toll in the region while preserving these important ecosystems.

## Introduction

Deforestation has been a major concern in much of the tropics because of its detrimental effect on biodiversity, atmospheric carbon emissions, regional weather patterns, among other ecosystem services [1]. The Brazilian Amazon in particular has received considerable attention because a large fraction of tropical forest clearing has occurred within this region [2]. This fact has prompted the creation of the world’s largest forest-conservation initiative to reduce emissions from deforestation and degradation (REDD+), with an initial pledge of up to $1 billion USD [3], and a commitment by the Brazilian government to reduce Amazon deforestation by 80% [4]. However, few conservation scientists seem to be aware that the Brazilian Amazon also plays an important role in terms of malaria cases and fatalities; almost half of the deaths attributed to this disease in the Americas occurred in Brazil [5], [6] and virtually all malaria cases in Brazil originate from the Brazilian Amazon [7], [8]. To reduce malaria morbidity and mortality in the region, multi-million dollar initiatives focused on malaria have also been created (e.g., $5 million USD/year from the Amazon Malaria Initiative [9]; and ∼$23 million USD from the Global Fund to Fight Aids, Tuberculosis, and Malaria [10]).

While it is generally agreed that environmental factors play an important role in malaria [11], there are mixed evidence regarding how land cover and deforestation affect malaria in the Amazon region. For instance, proximity to forest fringes [12]–[16] and land clearing [14], [17]–[24] have both been proposed to explain malaria vector presence, mosquito biting rate and malaria incidence. Yet, the exact role of these factors on malaria incidence has important implications regarding land use land cover (LULC) policies. Based on the evidence of higher malaria risk at recently deforested areas or in areas with active land clearing, it has been suggested that forest conservation can decrease disease burden [25]–[30]. Based on evidence of higher malaria risk when close to forest fringes, the opposite conclusion has been reached; it has been suggested that the long-term effect of land clearing is to increase the distance of humans to forest edges and thus decrease malaria risk [14],[31],[32].

These contrasting effects of deforestation have not been studied on a large spatial scale. Here we assess the magnitude of both of these malaria risk factors with a large malaria dataset (totaling ∼1.3 million positive malaria tests, gathered over ∼4.5 years and over a 4.5 million km^2^ region) and evaluate the public health consequences of current and future land use/land cover (LULC) scenarios.

## Methods

### Malaria Data

The malaria data were collected from January 2004 to August 2008 by the Brazilian malaria surveillance system [33] and are aggregated by month and health facility. A malaria case is defined as an individual that has fever and that has a positive *Plasmodium* spp. detection through microscopy [34]. To the best of our knowledge, this definition has been consistently used throughout the entire 2004–2008 period. Because there are no data on the exact location of each health facility, our approach was to subset the health facilities that are known to be in the urban area and use the spatial coordinates of the corresponding cities as proxies for their location. Determining the approximate location of these health facilities is important to adequately characterize the environmental risk factors to which individuals treated at these health facilities are exposed. We emphasize that despite being classified as urban areas, these are predominantly small cities (i.e., median population size equal to 14,000 people), often surrounded by a considerable area of forest (i.e., 22% of these cities had >50% of their catchment area covered by forests). The surrounding vegetation is critical because it is common for individuals to get infected in the surrounding area (e.g., while participating on selective timber logging, non-timber forest products collection, slash-and-burn agriculture, night fishing, hunting, mining, etc.) but to be diagnosed in the city [35],[36]. We further excluded cities that had less than two years of data because it would not be possible to estimate yearly and monthly city-specific random effects for these cities. The final dataset contained approximately half of the original malaria cases (∼1,300,000 cases) but covered a similar geographical area (96% of the counties in the original dataset) (a summary description of these data is available in Table S1).

### Catchment Area

We adopt a 20-km radius as the “catchment area” around each city and use the precipitation, deforestation rate, and forest cover estimates within this catchment area as our covariates. The size of this catchment area accounts for the malaria vector flight range [13],[21], population mobility to and from the surrounding vegetation, and the fact that malaria cases often arise from multiple urban health facilities within a particular city. The same radius has been used elsewhere as the area typically under urban influence in the Brazilian Amazon region [37]. Our results are robust to the use of different radii (i.e., 10, 20, and 30 km) (File S1 and Figure S2).

### Covariates

Population size comes from the 2007 Brazilian National Census, aggregated at the census tract level, made available by the Brazilian Institute of Geography and Statistics [38]. Our environmental covariates come from satellite imagery. We used annual forest cover and deforestation rate estimates from the Brazilian Space Agency derived from a semi-automated analysis of Landsat imagery [39]. Estimates of precipitation were derived from the Tropical Rainfall Measuring Mission data (‘3B43 Monthly 0.25×0.25 degree merged TRMM and other sources estimates’ product [40]), and average precipitation for a particular month was calculated over all pixels that fell within each catchment area. Based on these precipitation estimates, we also calculated a drought index that has been extensively used to characterize drought in the region [41]–[43]. We used a one month time lag for precipitation and drought index covariates based on the assumption that water affects the vector mainly through its breeding habitat. Therefore, changes in precipitation or drought should only affect infection risk in the following month since this is the minimum necessary time for the larva to become an adult mosquito, the adult to be infected and finally become infectious. Results did not change substantially when using a two month time lag (data not shown).

### Permutation Tests

To compare a particular outcome *X* (e.g., number of malaria cases per month, onwards simply malaria incidence) for cities with characteristic *c_1_* versus cities with characteristic *c_2_* (e.g., high vs. low forest cover), we first calculate the observed difference in means 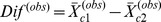. Then, we estimate the probability of an outcome equal or more extreme than the observed outcome under the null hypothesis (i.e., p-value) through a permutation test. To do this, we randomly assign these characteristics to the cities and calculate the simulated difference in means 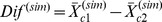. This was done 1,000 times, generating 1,000 values of 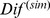. We estimate the p-value as 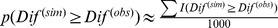, where I() is the indicator function that takes on the value of 1 if the condition is satisfied and zero otherwise.

### Regression Model Structure

We assessed the effect of forest cover (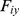, percent of catchment area) and annual deforestation rate (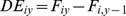, percent of catchment area per year) using a Bayesian hierarchical regression approach (*i* and *y* stand for city and year). We adjusted for potential confounder effect of climate variables on malaria risk, namely monthly precipitation 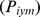 and a drought index 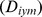 (*m* stands for month within year). All covariates were standardized (i.e., centered and divided by their standard deviation).

The number of malaria cases per month (i.e., malaria incidence) is modeled as an over-dispersed Poisson, given by:
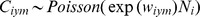
where 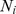 is the population size within the catchment area, and 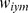 is given by:



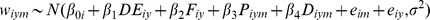
where 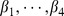 are fixed-effect regression parameters. Additional socio-economic-environmental covariates (e.g., proportion of migrants, age distribution, level of urbanization, gross domestic product, vector ecology, and proximity to large water bodies) tend to be relatively constant within the short time-frame of our malaria incidence dataset (∼4.5 years). Therefore, we control for these unspecified city-to-city differences using a city specific random intercept 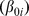. We also include a year-by-city 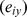 and a month-by-city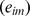 random effect. To complete the model specification, we adopt the usual assumptions regarding the distribution of random effects:



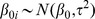


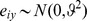


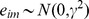
and we assume vague hyper-priors for the regression coefficients and variance parameters [44]:



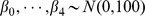


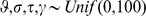



We used a Gibbs sampler to iteratively sample from each of the full conditional distributions. Because of conjugacy between likelihood and priors, almost all the parameters could be sampled directly [45]. The only parameters that could not be sampled directly were the 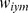, which were sampled with a Metropolis-within-Gibbs step.

We assessed model convergence by running three Markov Chain Monte Carlo (MCMC) chains with over-dispersed initial parameter values for 200,000 iterations. We discarded the first 10,000 iterations as burn-in and retained 500 iterations, systematically sampled from the remaining 190,000 iterations. We visually assessed convergence by overlaying trace plots of these three chains. We also assessed convergence by calculating the convergence statistic R suggested by Gelman and Rubin [46] (Table S2), where R values much greater than 1 indicate lack of convergence. Both, our plots and the convergence statistic R, suggest that convergence has been achieved.

To determine whether our model was over-fitting the data, we performed a validation exercise. In this exercise, we compared the out-of-sample predictive ability of our model versus simpler versions of it, either without the month-by-city random effects 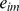 or without the year-by-city random effects 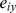. We fitted these models to 90% of the data and used the estimated parameters to predict the 10% of the data that was left out. The results from the validation exercise (data not shown) and the comparison between the data and the predictive posterior distribution for each city (Figure S1) revealed that our model had an adequate fit. Finally, a preliminary analysis indicated that the assumption of a linear relationship between LULC covariates and malaria incidence was adequate and revealed low temporal and spatial correlation (correlation on Pearson residuals <0.2), suggesting that additional nonlinear terms and parameters to model these correlations do not need to be included in our model. All analyses and figures were created using R [47].

#### Land Use Land Cover (LULC) future scenarios

To evaluate the long-term effect of conservation strategies in the Amazon basin, Soares-Filho et al. [48] simulated a governance (GOV) scenario and compared it to a business-as-usual (BAU) scenario, revealing that a substantial amount of deforestation (and its deleterious effects) could be avoided. These projections also allow us to evaluate the effect of future LULC trends on malaria. We estimated the ratio of the expected malaria incidence under the GOV scenario 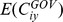 and under the BAU scenario 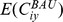 for each year and city. This ratio 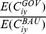 was calculated using the posterior distribution for the over-dispersed Poisson regression parameters, thus fully accounting for their uncertainty [49].

## Results

**Figure 1:**
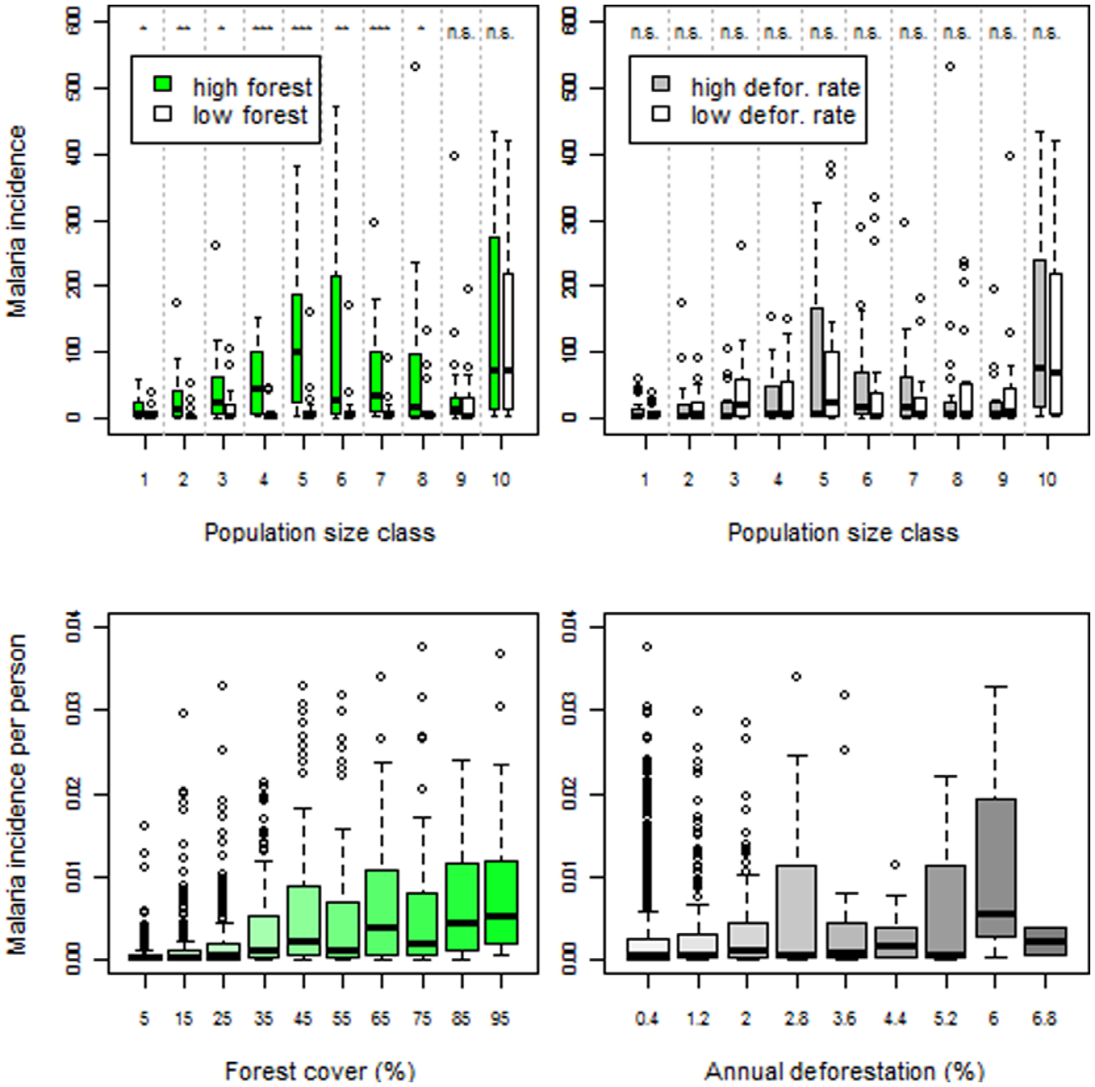
Malaria incidence is higher in areas with more forest cover whereas no clear pattern arises regarding deforestation rates. Upper panels: Data were stratified into 10 percentile population size classes and average number of malaria cases per month for each year and city was depicted. Within each size class, we compare cities with high (green box-plots) vs. low forest cover (white box-plots) (upper left panel); and cities with high (grey box-plots ) vs. low deforestation rate (white box-plots) (upper right panel). Cities with high forest cover (or high deforestation rates) are cities that have forest cover (or deforestation rate) higher than the median for that size class. ‘n.s’, ‘*’, ‘**’, and ‘***’ are non-significant (p>0.05), significant (0.01<p<0.05), very significant (0.001<p<0.01) and highly significant (p<0.001) difference in means, respectively, based on permutation tests. Lower panels: Mean number of malaria cases per month for each year and city divided by total population as a function of forest cover (lower left panel) and deforestation rate (lower right panel). Note: y-axes were truncated to enable a clearer depiction of the bulk of the data (i.e., less than 0.5% observations were excluded from these plots).

We find overwhelming evidence that areas with higher forest cover tend to be associated with higher malaria incidence whereas no clear pattern could be found for deforestation rates, when comparing cities with similar population sizes (upper panels in Figure 1). Similar evidence arises when analyzing malaria incidence per person across all cities (lower panels in Figure 1). Using a Hierarchical Bayesian regression, we show that although forest cover and deforestation rate were both positively associated with malaria incidence, forest cover effect was ∼25 times greater than that of deforestation rate (Table 1). As a result, the net effect of higher deforestation rates is to *decrease* malaria burden by decreasing forest cover (i.e., increasing the distance to forest fringes). We also find that the number of malaria cases was negatively correlated with precipitation and our drought index, suggesting that drier periods of the year tend to result in higher malaria incidence. These results were robust to alternative definitions of catchment area (File S1 and Figure S2). An alternative model specification, which explores changes in malaria incidence within each city (rather than within and between cities), revealed qualitatively similar results in relation to the LULC variables (File S1 and Table S3).

**Table 1:** Summary of regression parameter estimates.

Parameter	Covariate description	Mean	LCI	UCI
*β*_1_	Annual deforestation rate	0.04	0.00	0.08
*β*_2_	Forest Cover	1.03	0.87	1.20
*β*_3_	Precipitation	−0.07	−0.08	−0.05
*β*_4_	Drought index	−0.06	−0.07	−0.04

LCI and UCI: lower and upper limit of the 95% credible interval.

**Figure 2:**
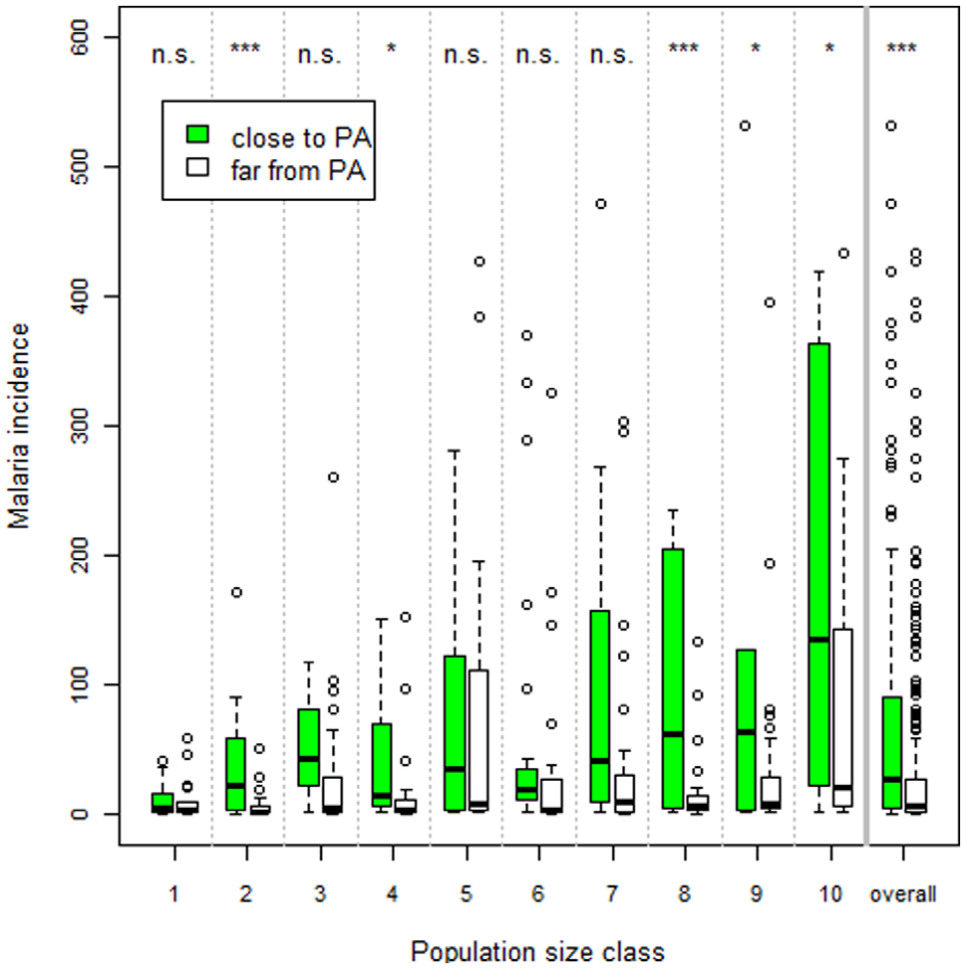
Malaria incidence tends to be higher for cities close to protected areas (PA’s). Data were stratified into 10 percentile population size classes and average number of malaria cases per month for each city was depicted. Within each size class, we compare cities close (green box-plots) vs. distant from PA’s (white box-plots). Cities close to PA’s (i.e., indigenous lands, state and federal parks) are those whose catchment area intersected one or more PA’s. ‘n.s’, ‘*’, ‘**’, and ‘***’ are non-significant (p>0.05), significant (0.01<p<0.05), very significant (0.001<p<0.01) and highly significant (p<0.001) difference in means, respectively, based on permutation tests.

**Figure 3:**
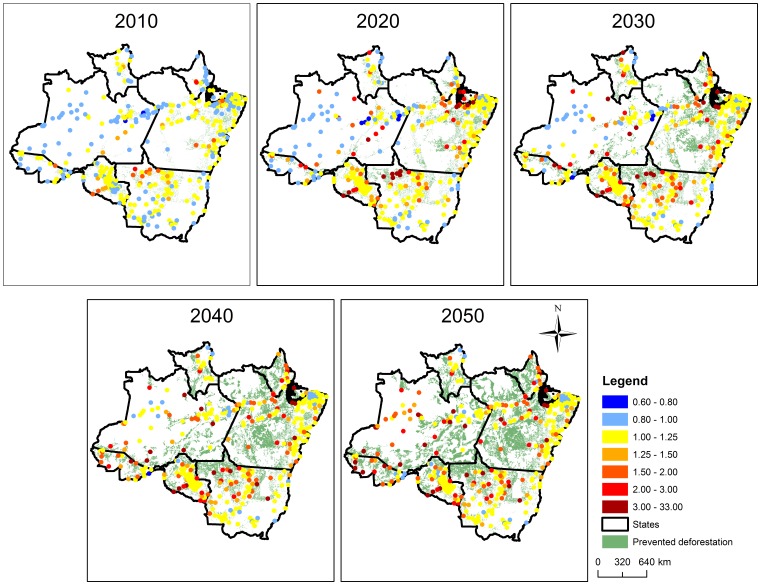
Predicted malaria incidence in urban health posts is higher in the governance scenario than in the business-as-usual scenario. Maps depict the ratio of the expected number of malaria cases per month for each year and city under the governance (GOV) and the business-as-usual (BAU) future LULC scenarios (i.e., 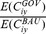), where values >1 indicate that the GOV scenario results in more malaria cases than the BAU scenario. Areas that were deforested in the BAU scenario but not in the GOV scenario (i.e., prevented deforestation) are depicted in the background for reference. Circles represent the cities in our original malaria dataset.

**Figure 4:**
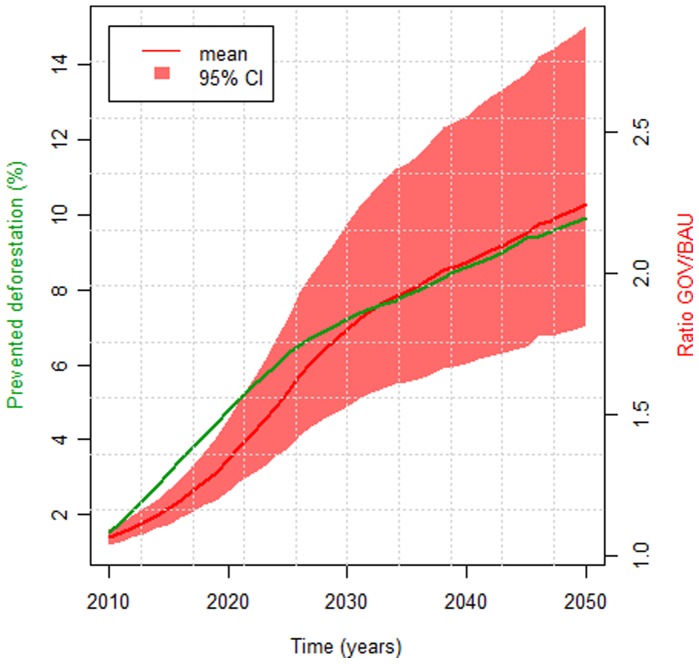
Malaria incidence increase at urban health posts in the governance scenario is predicted to be a direct consequence of prevented deforestation. We depict the relationship between future prevented deforestation under the governance scenario (green line), and the ratio of the expected malaria incidence for each year and city under the governance (GOV) and business-as-usual (BAU) future LULC scenarios (red line) (i.e., 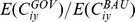), averaged across all cities. The red polygon represents the 95% credible interval of the average ratio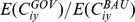.

These findings have important implications regarding LULC policies in the region. For instance, protected areas (PA’s) are a cornerstone of current conservation efforts, yet we are unaware of studies that discuss negative health impacts of these PA’s on the local population. A simple depiction of our malaria data suggest that cities close to protected areas (PA’s) tend to have higher malaria incidence than cities far from these PA’s (Figure 2) after controlling for population size, a likely consequence of higher forest cover in these areas. We also evaluated the long-term implications of our findings by comparing a future scenario with reduced deforestation (i.e., governance scenario - GOV) to a future business-as-usual (BAU) scenario. Using our regression parameter estimates, we find that cities with higher malaria incidence in the GOV versus the BAU scenario will initially tend to be concentrated in the south and east portion of the Brazilian Amazon (Figure 3), where roads slated for paving tend to be located. However, by 2050, almost all cities will tend to have higher malaria incidence. A summary of these results indicate that avoiding deforestation through better governance can substantially increase malaria incidence in urban health posts; an average of 10% of prevented deforestation resulted in an average 2-fold increase in the number of malaria cases per month by 2050 (Figure 4). These results raise concern regarding collateral public health effects of conservation policies.

## Discussion

We find that drier periods of the year tended to correlate with higher malaria incidence. Similar results have been attributed to decreased survival rate of adult mosquitoes [50] as well as larva being washed away in rivers [51] during the wet season. We refrain from further discussing these seasonal patterns here (we will address them in a separate paper) to focus our discussion on the LULC findings.

Malaria risk at frontier regions in the Amazon region is often observed to follow a peculiar time trajectory; in the early phases of human settlement, the number of malaria cases soars as naïve settlers arrive and engage in forest related extractive activities, living in precarious conditions. At later stages, as deforestation increases the distance of settlers to forest fringes and economic conditions improve, malaria risk tends to decrease through time [14]. Our findings regarding the LULC covariates agree with this later stage, suggesting that conservation efforts to decrease deforestation in places where people are already settled might inadvertently increase the number of malaria cases. Some would argue that conservation efforts will also decrease the amount of forest related extractive activities (e.g., fishing, hunting, extraction of non-timber forest products), thus decreasing malaria risk. We are skeptical; even if forest conservations efforts succeed in retaining forest cover, hunting and fishing is likely to continue to occur, even within protected areas [52]–[54].

We note that our finding directly contradicts the growing body of literature that suggests that forest conservation can decrease disease burden [25]–[30]. This literature often cite the study of Vittor et al. [21],[22] conducted in the Peruvian Amazon, as an example of how deforested areas favor the main malaria vector, *Anopheles darlingi*. However, similar entomological studies in the Brazilian Amazon region suggest the opposite pattern for the same vector species [15],[16],[55],[56], strongly supporting our results. This conflicting evidence might be due to distinct LULC patterns in these regions. In the Peruvian Amazon, swidden-fallow agriculture is the primary driver of deforestation and, as a result, deforested areas are often covered by shrubs and secondary vegetation growth [21],[22], whereas in the Brazilian Amazon, forests tend to be substituted by pasture and soy plantations [57].

Malaria occurring in urban areas is often attributed to poor housing and drainage conditions of slums [58],[59]. Furthermore, because slums are often located at the periphery of cities and thus closer to forests, this may give rise to a spurious association between forests and malaria incidence. We believe this hypothesis does not explain the malaria patterns we find in the Brazilian Amazon for several reasons. First, slums are rare in Brazilian Amazon cities because these cities are typically very small (i.e., as mentioned earlier, the median population size is 14,000) whereas slums tend to occur in bigger cities where a growing population in limited space gives origin to dense housing, often in hazardous sites. Using the Brazilian government census from 2010, we find that only 12% of the cities in our analysis had slums and that our results in Figure 1 do not change substantially after we exclude the cities with slums (data not shown). Second, the slum effect hypothesis predicts higher malaria incidence in bigger and poorer cities, contrary to the results depicted in Figure 1 and Figure S3. Finally, even after taking into account gender imbalances in the population of each city, we find that the average number of malaria cases per month per person tends to be higher in men than in women, a phenomenon that occurred in 96% of the cities in our dataset. This gender difference in malaria incidence agrees with our hypothesis that forest related activities in the surrounding areas, mostly conducted by men, are the cause of higher malaria rates rather than housing conditions.

Unfortunately, policies that have large effect on LULC in the region (e.g., road opening/paving, creation of rural settlement areas, and the establishment of protected areas) are traditionally perceived to lie in the realm of the Ministries of Environment, Infra-structure, Agriculture, and/or Energy, while the Ministry of Health typically focuses on the delivery of health services [7]. Similarly, global efforts are typically compartmentalized into conservation (e.g., REDD+) and public health (e.g., Roll Back Malaria and GFATM) initiatives. Few studies identify, or discuss how to address, trade-offs between these global efforts and governmental policies, probably because of the interdisciplinary nature of these trade-offs and the associated ethical issues. For instance, how can one reconcile potential conflicts between the Millenium Development Goals (e.g., goal of combating malaria and the goal of ensuring environmental sustainability)? Although we do not have an answer to this question, acknowledging that these tradeoffs exist is a critical first step towards finding a solution.

Current research and resulting policy recommendations regarding LULC in the Amazon ignore potential public health impacts. For instance, the most frequent policy action to decrease deforestation rates is to create protected areas [60]–[62]. Several studies suggest, however, that many of these protected areas are established in areas with small deforestation risk [61],[63], effectively averting few of the impacts of deforestation. These observations have resulted in recommendations to place these parks in areas more prone to deforestation [4],[63],[64], which often imply areas with larger human populations, disregarding the potential for increased malaria morbidity for the local population. Similarly, research acknowledging the negative effects of conservation efforts typically emphasize restrictions on agricultural development rather than the detrimental impact on public health [62],[65],[66].

One possible interpretation of our findings is that we are promoting deforestation. This is not the case. For instance, large-scale settlement projects in heavily forested areas have resulted in substantial deforestation *and* major malaria outbreaks in the past [14]. Here we argue that deforestation has both negative and positive effects in places where people are already settled, and that the knowledge of these effects is essential for proper LULC and public health planning, particularly in light of the recent ambitious REDD+ targets set by the Brazilian government and four of the Brazilian Amazon states [4]. If conservation efforts (e.g., REDD+) are to avoid this rapid land cover change and its associated adverse effects on several regional-global environmental services (e.g., atmospheric carbon emission, climate and biodiversity), these conservation efforts should, at a minimum, include proper malaria mitigation strategies (e.g., creation of more malaria detection and treatment outposts, distribution of long-lasting insecticidal bed nets, indoor residual spraying) to alleviate their local detrimental effects. Similarly, opportunity costs of reduced carbon emissions through conservation initiatives should take into account their local impact on malaria burden.

Our study has five important limitations. First, we do not take into account potential differences between cities in terms of main malaria vector species, vector ecology and infection efficiency. However, it is well known that collection of entomological data is extremely laborious [67] and therefore logistically impossible to collect over the same geographical scale as our malaria data. Yet, finding the same overall result over such a vast area by using a separate regression for each city (File S1 and Table S3) gives us confidence that our results are robust to these potential city-to-city differences. Second, in the absence of spatial coordinates of the individual health facilities, we rely on data from urban health facilities aggregated at the city level. Yet, we note that even if individual level data had been available, we would still not have been able to consider many individual-level factors that are known to be important for malaria risk (e.g., mobility, socio-economic status, housing conditions, and occupation) because only a few basic demographic characteristics, such as age and gender, are routinely collected by the malaria surveillance system. Third, in the absence of detailed information for a more accurate modeling of catchment area (e.g., network of unofficial roads [68], origin and mode of transportation of patients, treatment seeking behavior), we relied on relatively arbitrary radii to delimit the catchment area. Fortunately, our results were robust to changes in these radii. We emphasize that these three limitations are typical limitations of studies conducted over large geographical scales (e.g., the area of a single Brazilian Amazon state, Para, is equivalent to the combined area of France and Spain), illustrating the inherent tradeoff between local detail-rich studies, whose results may or may not be generalizable to a wider region, and large-scale detail-poor studies, which reveal broad scale relationships while ignoring many of the local complexities in malaria transmission. Importantly, while site-specific studies have been critical in shaping our knowledge regarding malaria in the region, they may be ill-suited to compare the effect of land clearing to the effect of forest cover because these covariates are often spatially correlated at this scale (i.e., land clearing often occurs in areas with high forest cover). On the other hand, over a large spatial scale, land clearing and forest cover are not highly correlated, allowing us to separately evaluate their effects.

The fourth limitation of our study is that, to avoid spatial extrapolation, our future scenario analysis only considers what would happen to malaria incidence in areas close to where humans are already settled (i.e., the vicinity of urban areas). In these areas, we assume that forests will give place to low intensity cattle ranching and soybean plantations [69],[70], thus increasing the distance between people and forest fringes. On the other hand, had we considered new human settlements (e.g., due to human migration to new agricultural frontiers), the BAU scenario might have indicated an initial higher malaria incidence due to an initial decrease in distance to forest fringes. Finally, as with any simulation study, our simulation results critically depend on the implicit assumption that everything else (e.g., age distribution, migratory patterns, patterns of natural resource extraction, climate, etc.) remains constant.

The clear pattern in the data (Figures 1 and 2), the consistency of our findings using alternative model specifications, and the evidence from detailed entomological and epidemiological studies in the region [12]–[16],[55] [56], suggest that the association between forest cover and malaria incidence we found is not spurious. Indeed, vegetation management has long been an important strategy to reduce the incidence of malaria [31]. Here we a) show that the effect of forest cover substantially outweighs the effect of deforestation rate (the often cited culprit for malaria in the region) and other climatic variables with a malaria dataset spanning an unprecedented geographical scale; and b) discuss the large-scale multi-sector (i.e., public health, development, and conservation) implications of these findings. Our results suggest caution regarding the widespread assumption that pristine ecosystems will always have beneficial effects for human health [25]–[30],[71]–[73]. We believe there are undoubtedly numerous ecosystem services from pristine environments; however, ecosystem disservices also exist and need to be acknowledged. Coordinated actions from apparently disparate science fields (e.g., epidemiologists and environmental scientists), government ministries (e.g., Ministry of Health and Ministry of Environment), and the ongoing multi-million dollar conservation and public health efforts in the region, will be required to decrease malaria toll in the region while preserving these important ecosystems.

## Supporting Information

Figure S1 **Comparison of the data (black line) and the 95% posterior predictive interval (red lines) for 20 randomly chosen cities.**
(JPEG)

Figure S2 **Posterior distribution of the main regression parameters with covariates and population size assessed using three different catchment area radii (10, 20, and 30 km).** A line at zero (dashed red line) was added for reference.
(JPEG)

Figure S3 **Gross domestic product is similar in cities with low and high forest cover.** Data were stratified into 10 percentile population size classes and average gross domestic product (GDP) for each year and city was depicted. Within each size class, we compare cities with high (green box-plots) vs. low forest cover (white box-plots). Cities with high forest cover are cities that have forest cover higher than the median for that size class. ‘n.s’, ‘*’, ‘**’, and ‘***’ are non-significant (p>0.05), significant (0.01<p<0.05), very significant (0.001<p<0.01) and highly significant (p<0.001) difference in means, respectively, based on permutation tests.
(TIFF)

Table S1 Summary description of the malaria dataset.
(DOC)

Table S2 Convergence statistic R [46] for the regression parameters (intercept and slopes for the different covariates) in the main model.
(DOC)

Table S3Summary statistics of the posterior distribution of the pooled forest cover effect 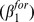
and deforestation rate effect 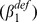 for the alternative model.(DOC)

File S1 Comparison of modeling results using different radii for the catchment area and description of the alternative model specification.
(DOC)
