# Supplementary figures and images for: Conservation Efforts May Increase Malaria Burden in the Brazilian Amazon
Source: PLoS One. 2013 Mar 6;8(3):e57519. doi: 10.1371/journal.pone.0057519 (PMC3590219; doi:10.1371/journal.pone.0057519)

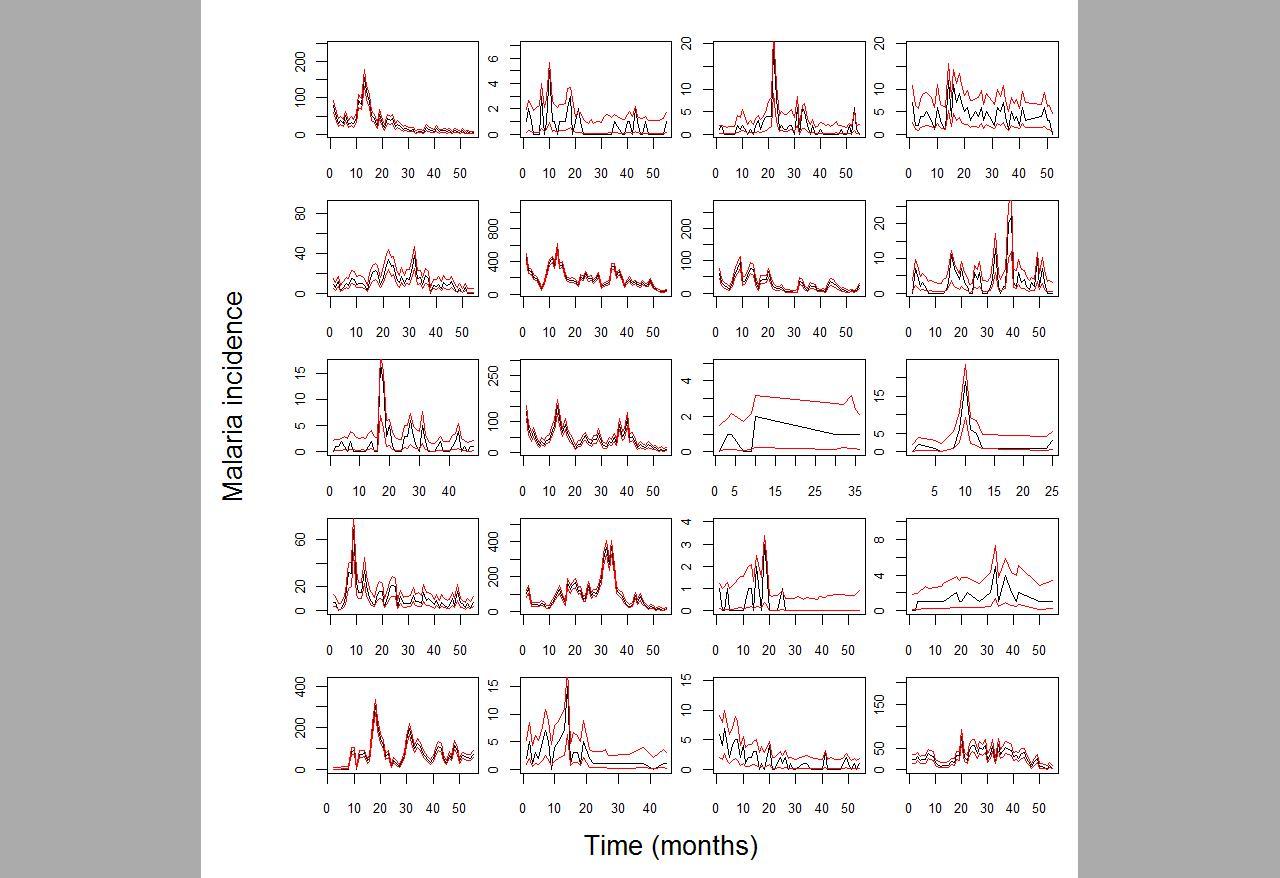

Supplement: Figure S1 — Comparison of the data (black line) and the 95% posterior predictive interval (red lines) for 20 randomly chosen cities. (JPEG) [file pone.0057519.s001.jpeg]

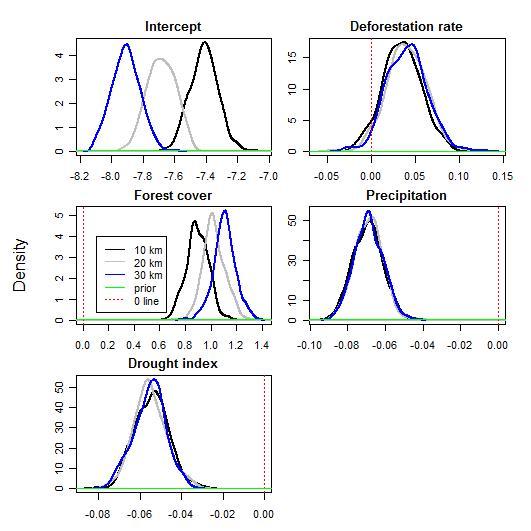

Supplement: Figure S2 — Posterior distribution of the main regression parameters with covariates and population size assessed using three different catchment area radii (10, 20, and 30 km). A line at zero (dashed red line) was added for reference. (JPEG) [file pone.0057519.s002.jpeg]

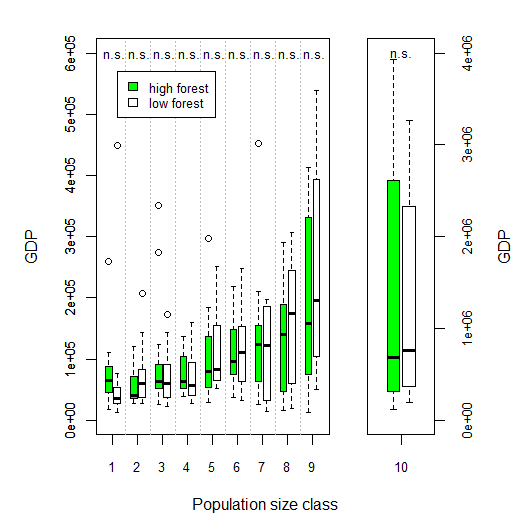

Supplement: Figure S3 — Gross domestic product is similar in cities with low and high forest cover. Data were stratified into 10 percentile population size classes and average gross domestic product (GDP) for each year and city was depicted. Within each size class, we compare cities with high (green box-plots) vs. low forest cover (white box-plots). Cities with high forest cover are cities that have forest cover higher than the median for that size class. ‘n.s’, ‘*’, ‘**’, and ‘***’ are non-significant (p>0.05), significant (0.01<p<0.05), very significant (0.001<p<0.01) and highly significant (p<0.001) difference in means, respectively, based on permutation tests. (TIFF) [file pone.0057519.s003.tiff]
