# Supplementary material for: Conservation Efforts May Increase Malaria Burden in the Brazilian Amazon
Source: PLoS One. 2013 Mar 6;8(3):e57519. doi: 10.1371/journal.pone.0057519 (PMC3590219; doi:10.1371/journal.pone.0057519)
Supplement: Table S1 — Summary description of the malaria dataset. (DOC) [file pone.0057519.s004.doc]

Table S1. Summary description of the malaria dataset.

| States | Number of Cities | Population  (in CA* per city) | Malaria cases  (in CA per month per city) | Forest cover  (% of CA per city) | Deforestation rate  (% of CA per city per year) | Precipitation  (mm in CA per month per city) |
| --- | --- | --- | --- | --- | --- | --- |
| Rondonia | 52 | 25617 | 96 | 0.24 | 0.008 | 155 |
| Acre | 22 | 26646 | 113 | 0.48 | 0.007 | 147 |
| Amazonas | 62 | 44780 | 157 | 0.60 | 0.001 | 193 |
| Roraima | 15 | 22010 | 72 | 0.36 | 0.005 | 177 |
| Para | 140 | 45625 | 32 | 0.23 | 0.006 | 231 |
| Amapa | 16 | 35783 | 87 | 0.34 | 0.002 | 237 |
| MatoGrosso | 94 | 32043 | 6 | 0.24 | 0.007 | 156 |

* CA: catchment area, defined as a 20 km buffer around each city

All columns, except for the states and number of cities, are averages of quantities assessed within each catchment area
