# Supplementary material for: Conservation Efforts May Increase Malaria Burden in the Brazilian Amazon
Source: PLoS One. 2013 Mar 6;8(3):e57519. doi: 10.1371/journal.pone.0057519 (PMC3590219; doi:10.1371/journal.pone.0057519)
Supplement: Table S2 — Convergence statistic R [46] for the regression parameters (intercept and slopes for the different covariates) in the main model. (DOC) [file pone.0057519.s005.doc]

Table S2. Convergence statistic R for the regression parameters (intercept and slopes for the different covariates) in the main model.

| Parameter | Point estimate | 97.5% quantile |
| --- | --- | --- |
| $\beta_{0}$ | 0.999 | 1.00 |
| $\beta_{1}$ | 1.002 | 1.01 |
| $\beta_{2}$ | 1.006 | 1.02 |
| $\beta_{3}$ | 0.999 | 1.00 |
| $\beta_{4}$ | 1.007 | 1.01 |
