# Supplementary material for: Conservation Efforts May Increase Malaria Burden in the Brazilian Amazon
Source: PLoS One. 2013 Mar 6;8(3):e57519. doi: 10.1371/journal.pone.0057519 (PMC3590219; doi:10.1371/journal.pone.0057519)
Supplement: Table S3 — Summary statistics of the posterior distribution of the pooled forest cover effect and deforestation rate effect for the alternative model. (DOC) [file pone.0057519.s006.doc]

Table S3. Summary statistics of the posterior distribution of the pooled forest cover effect $(\beta_{1}^{for})$and deforestation rate effect $(\beta_{1}^{def})$ for the alternative model.

| Parameter | Mean | LCI | UCI |
| --- | --- | --- | --- |
| $\beta_{1}^{def}$ | 0.09 | -0.02 | 0.20 |
| $\beta_{1}^{for}$ | 5.65 | 1.68 | 9.63 |

LCI and UCI: lower and upper limit of the 95% credible interval
