# Supplementary material for: Conservation Efforts May Increase Malaria Burden in the Brazilian Amazon
Source: PLoS One. 2013 Mar 6;8(3):e57519. doi: 10.1371/journal.pone.0057519 (PMC3590219; doi:10.1371/journal.pone.0057519)
Supplement: File S1 — Comparison of modeling results using different radii for the catchment area and description of the alternative model specification. (DOC) [file pone.0057519.s007.doc]

Supporting Information S1

Comparison of modeling results using different radii for the catchment area.

We evaluated how robust our results were to the definition of catchment area by quantifying our covariates and population size $N_{i}$ using different catchment area radii (i.e., 10, 20, and 30 km) and re-fitting our model. We find that the parameters that change the most were (intercept) and (forest cover effect) (Figure S2). However, these changes do not modify our overall conclusions, namely that the main determinant of malaria cases is forest cover, deforestation rate has a small but positive effect, and precipitation and drought index have a small but consistently negative effect.

Alternative model formulation.

The model in the main text was fitted using the pooled data from seven Brazilian Amazon states. Another approach is to use city-specific regressions and pool information from all these models, essentially exploring the within-city (rather than within-city and between-city) variability. The model is specified as follows:

$$C_{iym}\sim Poisson(\exp(w_{iym})N_{i})$$

where is the population size within the catchment area, and $w_{iym}$ is given by:

$$w_{iym}\sim N(\beta_{0i}+\beta_{1i}X_{iy},\sigma^{2})$$

We fitted two models, one where was deforestation rate and one where $X_{iy}$was forest cover. This model specification allows for city specific intercepts $\beta_{0i}$ and slopes $\beta_{1i}$. We can pool information from these parameters by assigning a random effect prior to them. Our priors were:

$$\beta_{0i}\sim N(\beta_{0},\tau^{2})$$

$$\beta_{1i}\sim N(\beta_{1},\gamma^{2})$$

$$\beta_{0},\beta_{1}\sim N(0,100)$$

$$\sigma,\tau,\gamma\sim Unif(0,100)$$

These statistical models revealed that the pooled forest cover slope was positive and significantly different from zero, and much larger than the pooled deforestation rate slope (Table S3).
